# Neuro-psychopharmacogenetics and Neurological Antecedents of Posttraumatic Stress Disorder: Unlocking the Mysteries of Resilience and Vulnerability

**DOI:** 10.2174/157015910793358123

**Published:** 2010-12

**Authors:** Abdalla Bowirrat, Thomas J.H. Chen, Kenneth Blum, Margaret Madigan, John A. Bailey, Amanda Lih Chuan Chen, B. William Downs, Eric R. Braverman, Shahien Radi, Roger L. Waite, Mallory Kerner, John Giordano, Siohban Morse, Marlene Oscar-Berman, Mark Gold

**Affiliations:** 1 Clinical Neuroscience & Population Genetics, and Department of Neurology, Ziv Medical Center, Safed, Israel; 2 Department of Occupational Safety and Health, Chang Jung Christian University, Taiwan, ROC; 3 Department of Psychiatry, University of Florida School of Medicine, Gainesville, FL, USA; 4 Department of Nutrigenomics and Personalized Medicine, Reward Deficiency Solutions LLC., San Diego, CA, USA; 5 Department of Engineering and Management of Advanced Technology, Chang Jung Christian University, Taiwan, ROC; 6 Department of Neurosurgery, Weill Cornell College of Medicine, New York, USA; 7 Department of Neurology, Ziv Government Medical Center, Safad, Israel; 8 Path Foundation, New York, USA; 9 Department of Holistic Medicine, G&G Holistic Addiction Treatment Center, North Miami Beach, Florida, USA; 10 Boston VA and Boston University School of Medicine, Boston Massachusetts, USA

**Keywords:** Post-traumatic Stress Disorder (PTSD), genes and environment, neurotransmitters, Reward Deficiency Syndrome (RDS).

## Abstract

**Background and Hypothesis:**

Although the biological underpinnings of immediate and protracted trauma-related responses are extremely complex, 40 years of research on humans and other mammals have demonstrated that trauma (particularly trauma early in the life cycle) has long-term effects on neurochemical responses to stressful events. These effects include the magnitude of the catecholamine response and the duration and extent of the cortisol response. In addition, a number of other biological systems are involved, including mesolimbic brain structures and various neurotransmitters. An understanding of the many genetic and environmental interactions contributing to stress-related responses will provide a diagnostic and treatment map, which will illuminate the vulnerability and resilience of individuals to Posttraumatic Stress Disorder (PTSD).

**Proposal and Conclusions:**

We propose that successful treatment of PTSD will involve preliminary genetic testing for specific polymorphisms. Early detection is especially important, because early treatment can improve outcome. When genetic testing reveals deficiencies, vulnerable individuals can be recommended for treatment with “body friendly” pharmacologic substances and/or nutrients. Results of our research suggest the following genes should be tested: serotoninergic, dopaminergic (DRD2, DAT, DBH), glucocorticoid, GABAergic (GABRB), apolipoprotein systems (APOE2), brain-derived neurotrophic factor, Monamine B, CNR1, Myo6, CRF-1 and CRF-2 receptors, and neuropeptide Y (NPY). Treatment in part should be developed that would up-regulate the expression of these genes to bring about a feeling of well being as well as a reduction in the frequency and intensity of the symptoms of PTSD.

## BACKGROUND

**Fig. (1):**
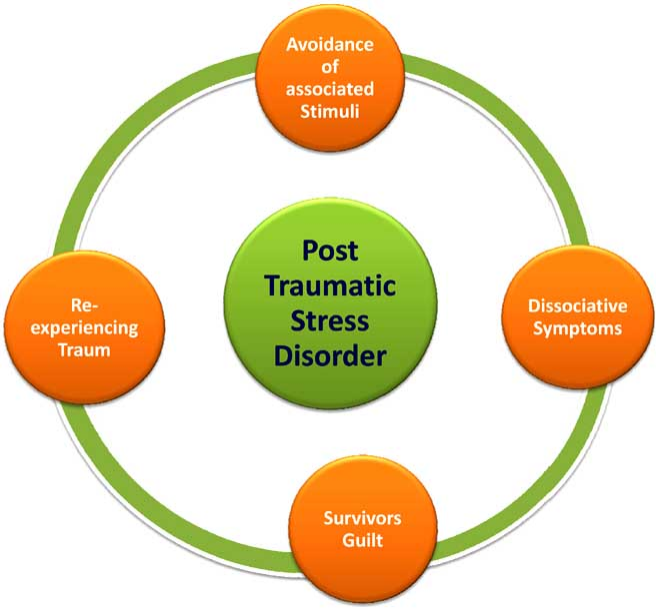
Characteristics of PTSD.

Post-Traumatic Stress Disorder (PTSD) is an anxiety disorder associated with exposure to a traumatic event outside the range of usual human experience, e.g., severe accidents, intense physical or sexual abuse, life-threatening medical conditions, combat, and natural disasters [1]. Common life events such as bereavement, chronic illness, and divorce rarely produce PTSD. The diagnosis of PTSD requires the presence of symptoms in each of three clusters including: re-experiencing the traumatic event through intrusive memories, flashbacks, and nightmares; avoidance and affective numbing; and hyperarousal (Fig. **1**). PTSD is a complex heterogeneous biopsychosocial disorder, and the stressors that produce PTSD are usually accompanied by intense fear, terror, and an overwhelming sense of helplessness [2]. Although the symptoms of PTSD can interfere with the ability to live a normal life and maintain normal relationships, its underlying pathophysiology remains ambiguous, despite sophisticated brain-imaging techniques and new genetic knowledge.

Psychological symptoms after trauma may include hyper vigilance and poor concentration. Flashbacks can occur consisting of images, sounds, smells, and feelings, causing the sufferer to lose touch with reality and repeatedly re-experience the traumatic incident. These often are triggered by ordinary occurrences such as a door slamming or the backfiring of a car. Furthermore, other symptoms such as insomnia, and an exaggerated startle response are common. Individuals with PTSD often re-experience the alarm phase response through their dreams and intrusive vivid recollections [2]. The symptoms often merge into anticipatory stress responses as the victims’ experience simultaneously mourning and fear [3]. 

Untreated PTSD causes significant disability and substantially increases medical utilization. It is well established that there is significant comorbidity with mood and anxiety disorders as well as Substance Use Disorder (SUD) [1, 4-6]. PTSD seems to affect all aspects of a person’s being, including body, mind, and spirit. Traumatic stressful experiences are stored in memory, perhaps in a cumulative manner along with other such life experiences, and the accumulation of traumatic life experiences can lead to increased vulnerability and decreased resiliency to further trauma [5]. Additionally, there are likely specific gene-environmental interactions that influence the vulnerability or resiliency to PTSD [7].

### Historical Perspective

Historically, symptoms associated with PTSD were reported as far back as 1900 BC by an Egyptian physician who described hysterical reactions to trauma [8]. About 15 centuries later, Hippocrates proposed a homeostasis theory to explain illness. That is, a stress reaction often results from the disruption of the balance or homeostasis of a system [9, 10]. The situation causing the stress reaction is defined as the "stressor". However it is the stress reaction and not the stressor that actually jeopardizes the homeostasis [5, 11]. Recent evidence suggests that post-traumatic stress responses result from a chemical imbalance of neurotransmitters. This seems to agree with the older views whereby stressful responses are tied to the homeostasis theory. Hysteria was also related to "traumatic reminiscences" a century ago [12]. At that time, Sigmund Freud's pupil, Kardiner described what later became recognized as PTSD symptoms [13]. 

PTSD has been recognized as a distinct psychiatric disorder since 1980. It is relatively common, with a lifetime prevalence of approximately 8% in the general population [1]. PTSD is often chronic, producing symptoms for at least one year in 50% of cases [5, 14]. Unfortunately, in many cases, these symptoms persist much longer [14, 15]. Treatment approaches to PTSD include individual therapy (cognitive, psychodynamic, or behavioral), group therapy, and medications such as antidepressants, anxiolytic drugs, and nutraceuticals [2, 16]. The treatment of PTSD is extremely challenging, requiring many years of therapy with variable results. Therefore, genetic screening for vulnerability and resilience may offer an important option for the prevention of PTSD and the amelioration of symptoms.

### Symptoms of PTSD

The three main clusters of symptoms that clinicians look for when diagnosing PTSD are re-experiencing, avoidance, and hyper-arousal (Fig. **1**) [1, 2, 17].

#### Re-experiencing symptoms

include intrusive memories of the traumatic event, recurrent distressing dreams about the traumatic event, and acting or feeling as if the traumatic event is reoccurring. Mental and physical distress often occurs when reminded of the traumatic event (e.g., on the anniversary of the traumatic event). Nightmares occur commonly in those suffering from PTSD. These nightmares can be frightening or highly emotional and are associated with disturbed sleep and daytime hyper-arousability. People with PTSD report awakening from dreams that involve reliving the trauma. The frequency of PTSD nightmares increases with the severity of the trauma. Unfortunately, these nightmares can persist long after the traumatic experience, and sometimes occur indefinitely [18]. 

The physiological symptoms following these frightening memories or nightmares can manifest as headaches, gastrointestinal complaints, immune system dysfunction, and chest pain. PTSD victims have an increased incidence of infections such as hepatitis and tuberculosis, muscle and skeletal diseases, hypertension, fibromyalgia, and circulatory disorders such as cerebrovascular disease, coronary artery disease, and hypertension [19, 20]. 

#### Avoidant symptoms

are unconscious possibly biophysical mechanisms that help to avoid any associations with the traumatic event. These symptoms manifest as emotional “numbing” of the sufferer’s general response to people and events. Avoidant symptoms often include avoiding thoughts, feelings, people, or situations associated with the traumatic event, an inability to recall aspects of the traumatic event, reduced interest or participation in significant activities, feeling disconnected from others, stress, showing a limited range of emotion, and having a foreshortened sense of future (e.g., not expecting to have a normal life span, marriage or career) [21-24]. 

#### Symptoms of increased arousal

may be similar to symptoms of anxiety or panic attacks. Increased arousal symptoms include being easily startled, difficulty concentrating, exaggerated watchfulness and wariness, irritability or outbursts of anger, and difficulty falling or staying asleep [25].

## THE NEUROCHEMISTRY OF PTSD

**Fig. (2):**
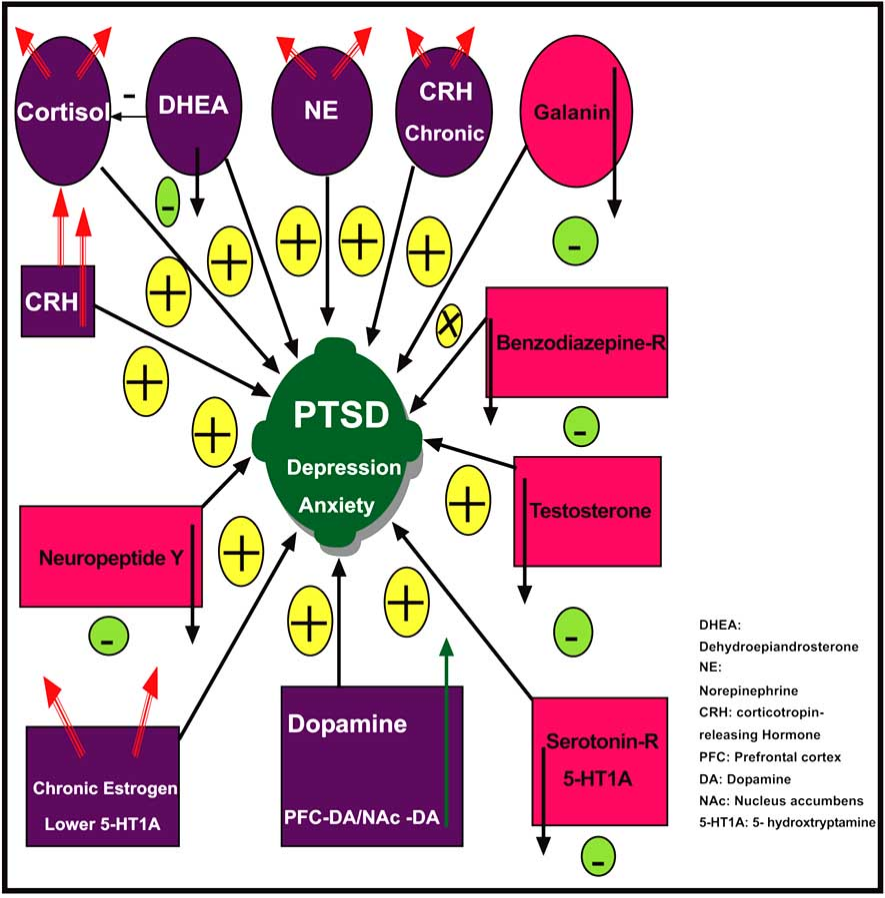
Neurochemical and hormonal responses in PTSD.

**Fig. (3):**
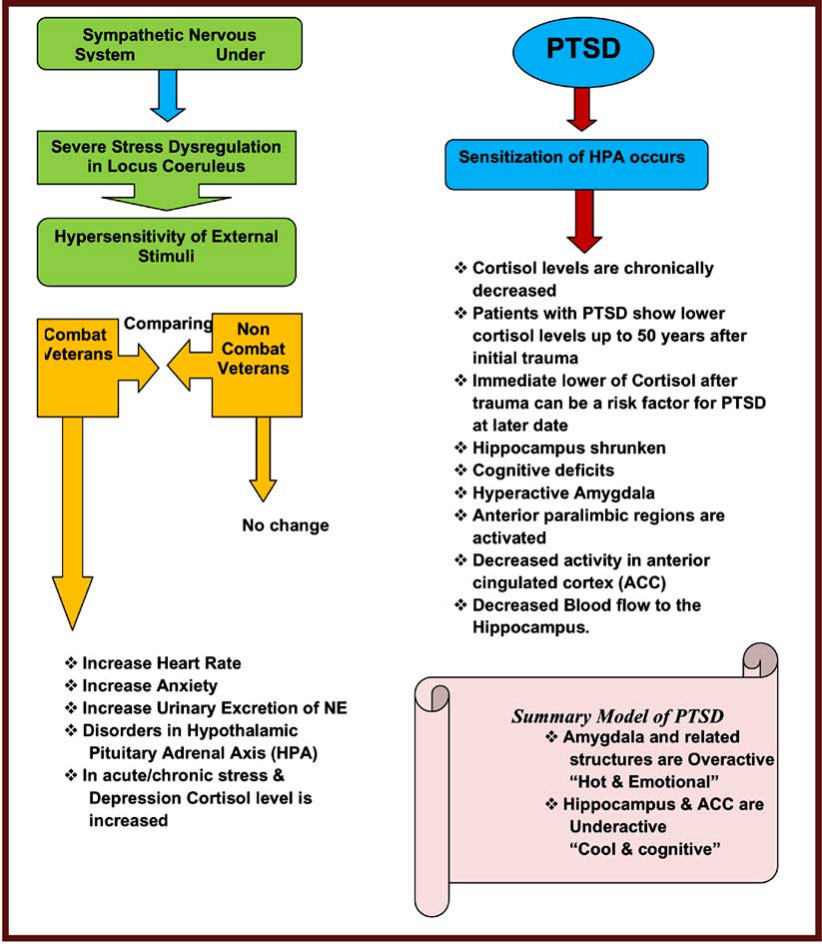
Legend: Summary of Neurobiology of PTSD.

**Fig. (4):**
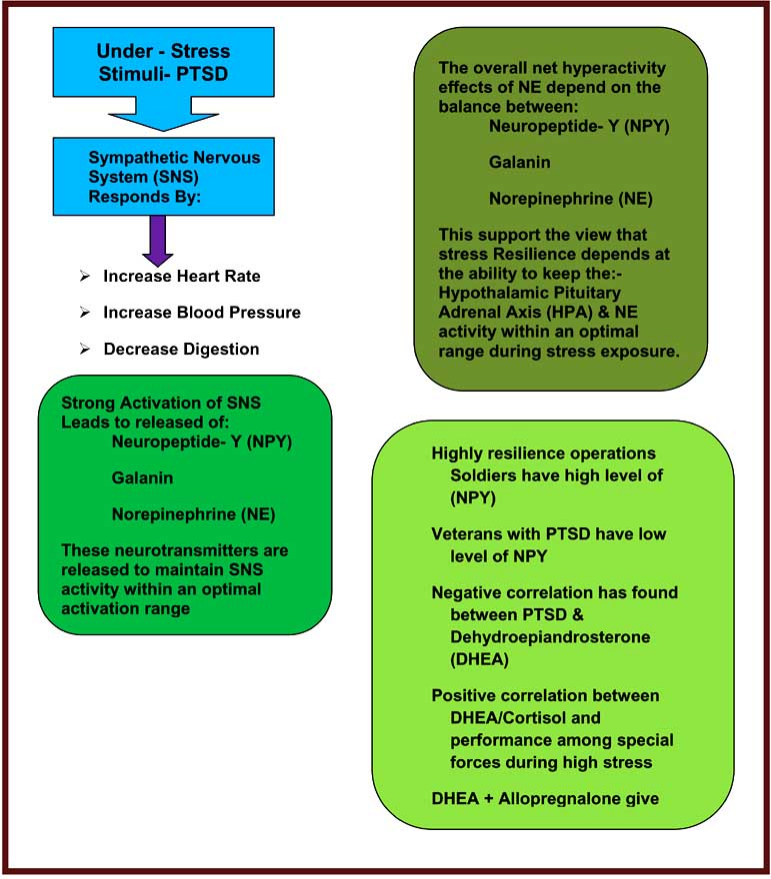
**PTSD and putative resilience mechanisms.** The sympathetic nervous system (SNS) responds to stressful events by increasing heart rate and blood pressure and by suppressing digestion. Additionally, the noradrenergic system often is dysregulated in PTSD. When the SNS is strongly activated, NPY, Galanin, and norepinephrine are released to maintain SNS activity within an optimal activation range. The overall net hyperactivity effects of norepinephrine depend on the balance between norepinephrine, NPY, and Galanin. This supports the view that stress resilience seems to be associated with an ability to keep the HPA-axis and norepinephrine activity within an optimal range during stress exposure.

In PTSD there are a number of systems that get activated for the purpose of survival (Fig. **2**). These include neurotransmitters such as dopamine, norepinephrine, epinephrine, opioid peptides, serotonin, GABA (gamma-aminobutyrate), glutamate, and acetylcholine, which are implicated in many psychological disorders. Additionally, there is evidence that neuropeptides such as the endorphins, somatostatin, vasopressin, and oxytocin play an important role. It is also likely that disruption occurs in the hypothalamic-pituitary-adrenal (HPA) axis [26]. The HPA system is activated by stressful stimuli, and the disruption that occurs with PTSD can be conceptualized as a kind of “false alarm”. There is evidence that dysfunction of the HPA system may produce hippocampal damage manifested as impaired memory [23]. It is hypothesized that a number of medications, such as selective serotonin reuptake inhibitors (SSRIs) may help to reverse neurochemical dysfunction in PTSD. It is as if these agents switch off many of the false alarms that characterize this condition (Figs. **3** and **4**). 

There is increasing evidence that sensitization and genetic defects of neurochemical systems are crucial pathophysiological characteristics of PTSD as evidenced by neuroimaging studies and genotyping [27,28]. The resultant sensitization may affect attention and decision-making and activate motor adaptive systems. Other systems are activated to counteract the crisis response. Once the danger has passed these neurochemical systems normalize, and homeostasis is restored. It has been proposed that two neuropeptides that are intimately involved in this mechanism are Neuropeptide-Y and galanin [29]. 

### Serotinergic Pathways

*Serotonin* is a neurotransmitter synthesized both in the central nervous system and in the gastrointestinal tract. It is believed to play an important role in depression, bipolar disorder, anxiety disorders, obsessive compulsive disorder, sexuality, appetite, sleep as well as pain. Serotonin helps to fight hunger, ameliorate depression and obsessive-compulsive symptoms, decrease pain in both fibromyalgia and migraine headaches, and promote sleep and weight loss. It was initially identified as a vasoconstrictor substance that increases tone in blood vessels hence the name "serotonin". Serotonin 5-hydroxytryptamine (5-HT) is synthesized from the amino acid tryptophan.

Taken orally, serotonin does not pass through the blood-brain barrier, and thus has no effect on serotonin levels in the brain. However, the amino acid tryptophan is capable of crossing the blood-brain barrier. Tryptophan is available as a dietary supplement although its ability to increase brain serotonin is still the subject of research and debate [16]. A variety of psychiatric medications increase brain serotonin levels. These include the monoamine oxidase inhibitors (MAOIs), tricyclic antidepressants (TCAs), and the selective serotonin re-uptake inhibitors (SSRIs). The MAOIs prevent the breakdown of serotonin and therefore increase its concentrations in the brain. However, MAOI therapy is associated with many adverse drug reactions and patients must follow a strict diet to avoid the hypertensive crisis that can be triggered by foods high in tyramine. Tricyclic antidepressants increase synaptic serotonin by inhibiting the re-uptake of both serotonin and norepinephrine. Like the MAO inhibitors, they have many side effects. More recently SSRIs have been developed. These have fewer side effects, drug interactions and are less toxic in the event of overdose.

Deficiency, or in some instances, excessive ingestion of certain vitamins and minerals can lead to altered serotonin levels through disruption of production or reuptake. Excessive increases in serotonin levels can produce a dangerous condition known as "Serotonin syndrome". The symptoms of serotonin syndrome may range from headaches, dizziness and vomiting, and progress to coma and cardiovascular collapse.

A number of studies suggest serotonergic involvement in PTSD. For example serotonin appears to play an important role in fear conditioning [30] and in stress-induced corticosteroid release [31]. Furthermore, in clinical studies, compared with controls, although those suffering from PTSD were shown to have greater affinity for paroxetine binding sites, they had a reduced number of binding sites. In addition, there was a relationship between pre-treatment paroxetine binding and clinical response to fluoxetine treatment [32-34]. Also, m-chlorophenylpiperazine (m-CPP), a serotonin agonist, provoked PTSD symptoms in patients with the disorder [35]. Finally, clinical overlaps between PTSD and depression, anxiety, impulsivity and aggression also suggest that serotonergic agents may be helpful in the treatment of this disorder [36].

### Catecholamine Pathways

*Catecholamines* are chemical compounds derived from the amino acid tyrosine that act as neurotransmitters. High catecholamine levels in blood are considered markers for stress. Catecholamines cause physiological changes that help prepare the body for physical activity, e.g., the fight-or-flight response. 

The catecholamines include compounds such as epinephrine (adrenaline), nor-epinephrine (noradrenaline), and dopamine. Epinephrine and nor-epinephrine are secreted by the adrenal medulla, and nor-epinephrine is also secreted by some nerve fibers. These substances prepare the body to meet emergencies such as cold, fatigue, and shock. 

#### Adrenaline (epinephrine)

acts as a neurotransmitter in the central nervous system and as a hormone in the blood circulation. It increases heart rate and stroke volume, dilates the pupils, and constricts arterioles in the skin and gut while dilating arterioles in leg muscles. It elevates blood sugar level by increasing the breakdown of glycogen and lipids. 

#### Noradrenaline (norepinephrine)

is primarily a peripheral sympathetic nervous system (SNS) neurotransmitter. In the brain, norepinephrine facilitates the formation of long-term memory and may preferentially facilitate the encoding of traumatic memory. It increases attention and may decrease impulsiveness. Along with epinephrine, norepinephrine plays an important role in the fight-or-flight response, by activating the SNS to increase heart rate, release energy from fat, and increase muscle readiness. Clinically, epinephrine is used to stimulate heart rate and contractility, as a pulmonary bronchodilator and to reduce intra-ocular pressure in glaucoma. 

#### Dopamine

is an intermediate in the synthesis of epinephrine. It has been shown that a deficiency in this neurotransmitter dopamine may result in difficulty coping with stressful conditions [37]. There is evidence that during the stressful events the neuronal levels of dopamine are depleted and when challenged with a dopamine agonist like cocaine an exaggerated response (sensitization) occurs in forebrain pathways. In addition, patients with PTSD sometimes demonstrate symptoms of hyper-vigilance and even paranoia that are likely to be mediated by the dopamine system [38].

### Opioid Pathways

The opioid system is involved in the release of dopamine in the nucleus accumbens [39] and also appears to play an important role in trauma by acting as an endogenous “pain-killer” [24]. As in the serotonergic system, the opioid system may become sensitized, with less intense shock producing subsequent analgesia. Not surprisingly, patients with PTSD often favor opioid substances of abuse [24, 39]. There is evidence that the opioid antagonist naloxone may reverse the analgesia induced by exposure to combat films. Moreover it is well known that stimulation of dopamine D2 receptors in the nucleus accumbens inhibits pain suggesting that the opioids are not working in isolation [40]. 

*Endorphins* are endogenous opioid polypeptide compounds. They are produced by the pituitary gland and the hypothalamus during strenuous exercise, excitement, pain, and orgasm, and like exogenous opioids such as morphine, fentanyl, and oxycodone produce analgesia and a sense of well being. Endorphins work as natural pain relievers, the effects of which may be enhanced by other medications as well as other drugs of abuse [41]. Endorphins are produced in response to stressful conditions and pain. They have been suggested as modulators of the so-called "runner's high" that athletes achieve with prolonged exercise [39]. At least 20 types of endorphins are known to exist. They are produced in the brain (primarily the hypothalamus and pituitary) and like exogenous opioids reduce our perception of pain. There is evidence that suggests that addiction to exercise, and possibly “thrill seeking behavior”, gambling etc, may produce an increase in dopamine and endogenous opioids, followed by down regulation in receptor density and changes in transport that result in a dependency on these activities, and symptoms of physical withdrawal upon cessation [42]. In addition to decreased feelings of pain, secretion of endorphins leads to feelings of euphoria, modulation of appetite, release of sex hormones, and enhancement of the immune response. Endorphin release varies among individuals. For example, two people who exercise at the same level or suffer the same degree of pain will not necessarily produce similar levels of endorphins. Sex and even certain foods, such as chocolate may increase endorphins. Furthermore, some studies have suggested that many of the positive effects produced by acupuncture, massage therapy, meditation, UV light, laughter and even the placebo effect may be mediated through endorphin release. Recently it has also been demonstrated that the release of endogenous opioids is associated with PTSD [24].

### Glucocorticoids

Cortisol is a steroid made in the adrenal glands is necessary in the functioning of almost every part of the body. It is the “stress hormone” produced in response to stressful conditions, both physical (illness, trauma, surgery, or temperature extremes) and psychological. It antagonizes insulin to promote the breakdown of carbohydrates, fatty acids, and proteins in order to produce the energy needed to respond to a potentially life threatening situation and ensure that the brain receives adequate energy resources. In the early stages of adrenal stress, cortisol levels will be elevated during the day and continue rising in the evening. Later, cortisol levels may rise and fall unevenly as the body struggles to balance itself against the effects produced by stressors such as caffeine, carbohydrates and vigorous exercise. In the middle stage, levels are abnormal and are especially elevated at night. In the advanced stages of adrenal dysregulation produced by prolonged stressful conditions, the adrenals may become exhausted from overwork, resulting in subnormal cortisol levels (hypoadrenia). Clinically, adrenal dysfunction may only be evident in its extreme stages e.g. Cushing’s disease or Addison’s disease. 

Sustained high cortisol levels may produce many adverse effects. For example, destruction of healthy muscle and bone can occur. Healing and cell replacement can be delayed. Metabolism can be impaired resulting in increased blood sugar, diabetes, increased abdominal fat, heart disease, and suppressed endocrine and immune function. By-products of cortisol depress brain activity and produce sedation. Cortisol itself can block serotonin, possibly producing or contributing to feelings of depression. Furthermore, adrenal dysfunction may be a factor in many other related conditions, including fibromyalgia, hypothyroidism, chronic fatigue syndrome, arthritis, and menstrual difficulties. Adrenal fatigue may also produce insomnia, and daytime somnolence. Long-term exposure to cortisol may produce hippocampal damage resulting in impaired learning as well as mood dysregulation. Conversely, short-term exposure of cortisol may actually help to implant succinct "flashbulb memories". In addition there is evidence that elevated cortisol can potentially damage other parts of the brain that regulate emotions, impulsive behavior, arousal, and attention. 

Cortisol excess can produce overt physical changes that are easy to detect. These include high blood sugar, weight gain, hypertension, and dementia. The effect of elevated cortisol on mental function is subtler and occurs at lower levels. These levels are frequently abnormal in patients with mood disorders. This suggests that cortisol may affect as well as be affected by one’s mental state. 

Normally when cortisol is present in excessive amounts, a negative feedback system is set into place. High levels of cortisol inhibit the production of hypothalamic Corticotropin Releasing Factor (CRF), which results in feedback inhibition of adrenal cortico-trophic hormone (ACTH) secretion from the pituitary. However, animal studies suggest that this feedback system can break down in response to chronic stress. Finally, in a recent study, the administration of exogenous cortisol to elderly PTSD patients resulted in increased glucose brain metabolism [43].

Alterations in neurotransmitter and hormone activity involving serotonin, natural opiates, catecholamines, and cortisol result in the wide range of objective and subjective PTSD symptoms [44]. The intrusive symptoms of PTSD (sudden and frightening thoughts, memories, flashbacks, and nightmares) have been linked to high levels of CRF [45]. An elevation of CRF may be conceptualized as an ignition switch for the stress responses. This activation and the subsequent release of epinephrine may have a direct effect on the formation of memories. The avoidance cluster of PTSD symptoms (emotional numbing, sleep disturbances, depression, and the use of “escape” drugs such as alcohol and illicit substances) have been linked to elevated levels of endogenous opioids, which are produced in the alarm phase to temporarily mask pain. The hyper-arousal group of symptoms (anxiety disorders, irritability, anger outburst, hyper-vigilance, dizziness, sudden startle reflex, and sometimes feelings of intense survivor guilt) has been linked to exaggerated CRF levels and heightened SNS activity. 

### Conjectured Neurotransmitters and Treatment Possibilities

There is evidence that many of the neurobiological changes found in PTSD can be correlated with symptom clusters [44]. For example, noradrenergic sensitization may be responsible for hyper-arousal symptoms [35] and opioid dysfunction may underlie the psychological numbing symptoms seen in PTSD. Furthermore, dopaminergic dysfunction may mediate symptoms of hyper-vigilance and paranoia [34], while Cortisol mediated damage to the hippocampus may be responsible for problems with memory and recall. Such a schema, though undoubtedly simplistic, may also lead to a heuristic approach to the pharmacotherapy and nutritional therapy for PTSD [44]. For example, tricyclic antidepressants, which act on the noradrenergic system, may be useful in reversing hyper-arousal symptoms. Similarly, clonidine, an alpha-2-agonist, which decreases noradrenergic output, may also be useful in ameliorating symptoms related to sympathetic hyper-arousal. Likewise, medications that block dopamine receptors may be useful for patients with psychotic or near-psychotic symptoms. Though opioid receptor blockers, such as naltrexone have not been well studied in PTSD, they may turn out to have utility. We now know that serotonin interacts with a range of other neurotransmitters. This may account in part for the promising effects of the serotonin re-uptake inhibitors in PTSD. Moreover, evidence is emerging which suggest that nutraceutical induced dopaminergic activation based on specific gene polymorphisms may play an important future role [16].

## THE NEUROANATOMY OF PTSD

On a macroscopic level, recent research suggests that the signs and symptoms of PTSD may also have anatomic structural correlations. For example, we know that sensory input, memory formation and stress response mechanisms are affected in patients with PTSD. The regions of the brain involved in memory processing that have been implicated include the hippocampus, amygdala, and frontal cortex. Likewise, functional abnormalities within the thalamus, hypothalamus, and locus coeruleus may be responsible for the heightened stress response (see Table **1**). 

**Fig. (5):**
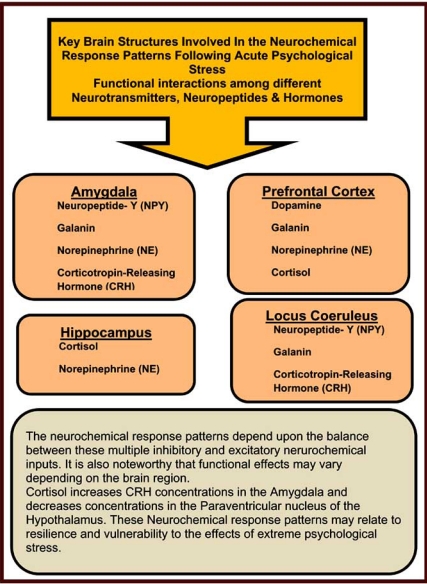
**Legend: Neurobiology, Neurochemical & Physiology of traumatic stress.** This figure illustrates some of the key brain structures involved in the neurochemical response patterns following acute psychological stress. The functional interactions among the different neurotransmitters; neuropeptides, and hormones are emphasized. It is apparent the functional status of brain regions such as the amygdala (neuropeptide Y, galanin, corticotropin-releasing hormone [CRH], cortisol, and norepinephrine), hippocampus (cortisol and norepinephrine), locus coeruleus (neuropeptide Y, galanin, and CRH), and prefrontal cortex (dopamine, norepinephrine, galanin, and cortisol) will depend upon the balance among multiple inhibitory and excitatory neurochemical inputs. It is also noteworthy that functional effects may vary depending on the brain region. Cortisol increases CRH concentrations in the amygdala and decreases concentrations in the paraventricular nucleus of the hypothalamus. As described in the text, these neurochemical response patterns may relate to resilience and vulnerability and to the effects of extreme psychological stress.

People with PTSD often show structural and functional abnormalities of the brain. The primary regions that have been implicated include the limbic system and prefrontal cortex (see Fig. **5**) [46]. 

Normally, humans respond to stressful conditions with adaptive neurochemical changes that enable us to fight, flee, or freeze. These changes are extremely rapid and occur the moment our brain recognizes a life-threatening event. The areas of the brain that are involved in the control and expression of mood and emotion, processing and storage of recent memories, and in the control of appetite and emotional responses to food are known collectively as the limbic system.

### Limbic System

The limbic system consists of several structures located around the thalamus (see Fig. **5**) and affects the endocrine and autonomic nervous system. It is responsible for our survival instincts and reflexes, as well as regulating autonomic nervous system responses to stress responses and relaxation. The structures in the limbic system indicate composite substructures of the forebrain that is variously defined on the basis of function and connectivity. These structures are comprised of the cingulated gyrus, parahippocampal gyrus, hippocampal formation, septal nuclei, amygdala, hypothalamus, epithalamus, as well as the anterior nuclear group of the thalamus and portions of the basal ganglia. 

Three of the structures that are found in the llimbic system are of particular importance in PTSD. These are the amygdala, hippocampus, and the hypothalamus. 

### Amygdala

The amygdala receives inputs from the thalamus and the cortex, and sends efferents to the brainstem, hypothalamus, and striatum. It is likely that these circuits play an important role in responding to threatening information *via* the autonomic, neuroendocrine, and motor systems. Recently, preclinical studies showed that amygdala circuits are involved in fear conditioning and extinction [44]. For example, when a rat is shocked each time it hears a particular noise, it develops a rapid fear-response to that noise (conditioning), controlled through the thalamo-amygdala pathway. If the rat is repeatedly exposed to the same noise without being shocked, it will gradually lose the fear as behavioral extinction occurs. Evidence suggests that this involves the slower cortical-amygdala pathways, which inhibit the earlier associations.

The amygdala is linked to responses like fear, pleasure, feelings of punishment, and awareness of behavior. It receives input from sensory systems, and it attaches emotional meaning to these sensations. It also signals other parts of the brain when danger is perceived and sends outputs to the hypothalamus, activating the SNS. In turn, this triggers stress catecholamine release, thereby initiating the flight-or-fight response. There is evidence that conditions such as PTSD, anxiety, autism, depression, narcoleptcy, and schizophrenia are linked to amygdala dysfunction secondary to neurotransmitter imbalance, structural damage, or developmental problems. Several neuroimaging studies have demonstrated increased activation of the amygdala in PTSD, though this finding is not always consistent [47, 48]. One study demonstrated an association between a history of neglect and decreased amygdala activation [49]. This decreased activation may actually serve an adaptive function under certain environmental conditions by allowing for continued functioning in situations characterized by ongoing threat. However, immobilization defenses such as avoidance, freezing, display of submissive behaviors, etc. may occur, and be maladaptive. Furthermore, it is not uncommon for persons with an abuse history to seek out abusive relationships, part of what psychoanalysts refer to as the repetition compulsion, without being consciously aware of, or by minimizing the dangers involved in such relationships [50].

### Hippocampus

Anatomically, the hippocampus resides in the temporal lobes, and sends and receives inputs from the amygdala and the cortex. This action seems to be mediated in part by cortisol through its action on hippocampal glucocorticoid receptors [44]. The hippocampus is the part of the brain that is concerned with the formation, organization, and storage of memories. It is believed that the hippocampus is involved in the generation and recollection of episodic memories, particularly memories for personal experiences and facts, and it links emotions and sensory inputs (e.g., sights, smells, and sounds) in memory. The hippocampus ceases to play a crucial role in the retention of the memory after a period of consolidation, such as when we've mastered a new skill. Damage to the hippocampus usually results in profound difficulties in forming new memories, while sparing some aspects of memory such as the ability to learn new skills (e.g., playing a musical instrument). The hippocampus plays an important role in memory, and these circuits may be involved in mediating explicit memories of traumatic events as well as learned responses to a constellation of cues ("contextual fear conditioning"). Furthermore, preclinical studies demonstrated that cell death of hippocampal neurons followed by hippocampal shrinkage may occur in animals after exposure to chronic stressful conditions. This reaction may be mediated in part by hippocampal glucocorticoid receptors [51]. 

Almost a decade ago, research confirmed the involvement of the hippocampus and amygdala in PTSD [47]. More recently, positron emission tomography (PET) studies showed that veterans with PTSD demonstrated increased right amygdala activity when exposed to combat movies [52]. Structural magnetic resonance imaging (MRI) studies have shown that male combat veterans, women survivors of childhood sexual abuse, as well as persons who have been diagnosed with major depressive, bipolar and schizophrenic disorders, have decreased hippocampal volumes [53]. Given the function of the hippocampus, it is not surprising that in many of these studies, this change correlated well with memory deficits. 

PTSD involves memories of traumatic events. These differ from other kinds of memories in several ways. For example, traumatic memories may persist over many decades, are easily triggered by reminders associated with the traumatic events, and their affect-laden quality can make them difficult to verbalize. Memory in PTSD patients is also characterized by several different kinds of impairments. These include not being able to recall specific aspects of the traumatic events. Furthermore, these memories can be fragmented and difficult to place in chronological context. These clinical symptoms are entirely consistent with current evidence of dysfunction involving the amygdala and hippocampus, structures that are thought to play key roles in the "emotional memory system" of the brain [18]. 

Functional neuroimaging of the hippocampus clearly demonstrated that it performs a vital role in detection of novelty, episodic memory consolidation and recall, and as a behavioral inhibition system. As previously mentioned, there is consistent evidence from MRI volumetric studies that hippocampal volume is reduced in PTSD. This atrophy of the hippocampus is thought to represent actual decreased neuronal density. On the other hand, there are also studies that suggest that these structural hippocampal changes may be a part of the whole brain and generalized white matter atrophy that can often be found in those suffering from PTSD [48]. Several reports suggest that stress related glucocorticoids may be involved in the atrophy of hippocampal CA3 pyramidal cells. The CA3 region of the hippocampus is a major target for glucocorticoids in the brain, as it plays an important role in regulating the pituitary-adrenocortical response to stressful conditions. Glucocorticoid induced atrophy would appear to require prolonged or repeated bursts of glucocorticoid excess. However, basal glucocorticoid levels have been shown to be lower than normal in PTSD, perhaps due to enhanced adrenocortical sensitivity to feedback regulation [54]. Furthermore, Yehuda *et al*., found that dexamethasone administration in PTSD enhanced suppression of cortisol [55].

The cognitive correlates of the hippocampal damage often found in PTSD are an impairment of episodic memory and novelty detection. This may disrupt executive function, resulting in uncertainty, distraction, and anxiety [23]. Press *et al*. reported a 49% reduction of hippocampal volume in amnesic patients, with virtually normal parahippocampal volumes. These patients showed severe deficits of verbal and non-verbal memory, while performing normally on various other tasks [23]. 

Since Bremner *et al*., presented evidence that the hippocampus of patients suffering from PTSD was reduced in size in 1995 [56], many other studies have looked at hippocampal volume. Although hippocampal atrophy has not been observed in every single group of patients, it is widely accepted that traumatic or prolonged stress can potentially alter the size of the hippocampus. Studies using animal models have also demonstrated that severe stress can alter hippocampal structure and function [53, 57-59]. 

A number of studies have now demonstrated the involvement of the hippocampus in chronic PTSD. Two studies have examined the hippocampus without finding volumetric changes. The first study demonstrated the absence of hippocampal atrophy prior to the onset of PTSD [60]. Further, after single-event trauma, hippocampal abnormalities did not occur within six months. The second study [10] demonstrated smaller cerebral volumes and corpus callosum measures but no hippocampal changes in a group of maltreated children.

Sapolsky [61] has described the sensitivity of hippocampal formation to severe, chronic traumatic stress and perhaps also to elevated glucocorticoids and/or excitatory amino acids, yet many other disorders such as bipolar disorder, schizophrenia, alcohol misuse and dementia are also associated with hippocampal atrophy [62]. The lateralization of hippocampal damage is variable. One possible explanation for this could be changing vulnerability of the hippocampus to stress-induced damage at different developmental stages. The possibility that small hippocampi represent a predisposition to PTSD was not supported by the one study examining acute PTSD [60] because no change in volume manifested acutely or within the first six months. Furthermore, Bremner *et al*. demonstrated no volume difference between early-onset (before age 8 years) and late-onset (age 8 years or older) abuse [49]. His group also found an 8% reduction of right hippocampal volume in Vietnam veterans with PTSD. This change was associated with deficits in verbal short-term memory [WMS] [56].

Gurvitis *et al*. [63] reported bilateral hippocampal volume reduction in combat PTSD after controlling for age and whole brain volume. His group noted that the decrease was greater on the left (26%) than the right (22%), especially after controlling for excessive alcohol consumption and combat exposure. The decreased hippocampal volume was strongly related to combat exposure, PTSD symptom severity, measures of attention, and memory scores. There were no differences in the size of the intra-cranial cavity, whole brain, ventricles, ventricle to brain ratio, or the amygdala (although, the right amygdala was somewhat larger in PTSD patients than in controls). 

Furthermore, Bremner *et al*. [49] reported a 12% reduction in the size of the left hippocampus in adult survivors of chronic childhood abuse (seven to 15 years of physical/sexual/emotional abuse). Ten percent of this reduction was related solely to PTSD after sex, age, education, and alcohol abuse were controlled using linear regression. There was also a statistically insignificant 5% reduction in right hippocampal size and no differences in caudate or amygdala volume. However, there was a larger left temporal lobe in these patients as compared to controls. The reduced left hippocampal volume was not correlated with significant verbal memory impairments (immediate and delayed recall and retention), trauma onset or duration, years since trauma cessation, or PTSD symptom severity. 

Trauma early in life may damage hippocampal structures, but adaptive neuroplastic changes could potentially allocate verbal memory processes to other structures, thereby diminishing the correlation between hippocampal volume and verbal memory impairment. Larger left temporal lobe size could account for better visual performance in these patients. This may be an adaptation to a loss of verbal capacity. Stein *et al*. [64] also reported a 5% reduction of left hippocampus in women survivors of severe childhood sexual abuse. Canive *et al*. [65] reported focal white matter lesions in eight of 42 combat related PTSD patients using a FLAIR MRI acquisition. These lesions were located either in periventricular regions or near the white/grey cortical junctions, and were not associated with symptom severity or comorbid depression or alcohol abuse. The hippocampus may be unique in the brain in its ability to regenerate neurons [66], with agents such as phenytoin (anti-epileptic drugs) potentially able to reverse stress-activated hippocampal atrophy [67]. Moreover, given our emergent knowledge on neurogenesis, i.e., the birth of new cells in the hippocampus, and the reversibility of cortisol-induced neuropathology, there is reason to believe that treatment can be effective in helping this condition reverse itself [45].

### Hypothalamus

Brain function depends on neurochemicals and substances secreted by the endocrine system. The endocrine system uses hormones as chemical messengers. These hormones circulate from one organ to another through the bloodstream. The target or destination organ interprets the hormonal signals and acts on them accordingly. The endocrine and nervous systems are linked by the hypothalamus (a centrally located 'switching station' within the brain). The hypothalamus is an exceptionally complex region with multiple components that control many different body functions including the regulation of blood pressure, hunger, immune responses, body temperature, and maternal behavior.

The hypothalamus also is considered to be part of the limbic system. It mediates between the nervous system and the endocrine system, and helps to regulate blood pressure, heart rate, hunger, thirst, sexual arousal, and the sleep/wake cycle. The hypothalamus secretes releasing hormones to the nearby pituitary in response to nervous system stimuli including smell, taste, pain, and emotions. For example, stressful conditions, cold, heat, and other stimuli cause a release of CRF from the hypothalamus. The CRF then travels to the pituitary. The pituitary is called the "master gland" because it produces hormones that regulate a wide variety of bodily activities, including growth, blood pressure, pregnancy, sexual function, metabolism, and water balance in the body. 

Through the HPA axis, the hippocampus and the amygdala activate the hypothalamus, which then releases CRF. When CRF then activates the pituitary gland, ACTH is released. Finally ACTH stimulates the adrenal glands to release the hormone, cortisol. The level of cortisol is proportional in magnitude to stress level. Using a negative feedback loop, it then inhibits the SNS and attenuates the HPA axis. The enhanced activity of the SNS and the role of HPA in the mechanisms responsible for memory impairment, phobic reactions, hyperactivity, obsession, and sleep disturbances that are associated with PTSD have been reviewed [17] and remain a source of intense investigation.

### Thalamus

All sensory information, with the exception of olfaction, is communicated through the thalamus to the cerebral cortex. Krystal *et al.* [68] theorized that the thalamus serves as a gateway that modulates the access of sensory information to the cortex, amygdala, and hippocampus. Lanius and colleagues [21] have hypothesized that the thalamus is important in the interaction between cognition, affects and behavior. These researchers, along with others, have reported thalamic dysfunction in persons diagnosed with PTSD. This dysfunction may disrupt the relay and integration of sensory information to the neo-cortex and limbic system. Thalamic dysfunction may, therefore, underlie the flashbacks observed in PTSD as well as the inability to integrate traumatic memories into the present. 

The inability to integrate traumatic memories into the present may be related to disruptions in thalamus-mediated temporal binding. The latter refers to the 40 Hz oscillations in the thalamus, which results in synchronous activity of “reentrant thalamocortical loops”. During alert mental activity, nerve cells in the thalamus oscillate at a frequency of 40 HZ. The connections of thalamic cells with cells in the cortex have been proposed to lead to similar frequencies of cortical cell oscillations, thus creating reentrant thalamocortical feedback loops. Temporal binding has been proposed to be a coherent event that binds components of internal and external realities into a single construct “the self”. In the absence of temporal binding, individuals cannot integrate the totality of what is happening to them into personal memory and identity. Thus, PTSD has been proposed to be a disorder of “thalamocortical dysrhythmia” whereby upper brain structures would become temporarily disconnected from lower structures each time the traumatic memory is accessed, interfering with processing and integration [21, 69].

### Orbitofrontal Cortex

The orbitofrontal cortex is a part of the frontal lobes lying just above the orbit of the eyes. It receives input from adjoining cortical and subcortical areas, including the dorsomedial thalamus, temporal cortex, ventral tegmental area, olfactory lobe, and the amygdala. It has efferents to both cortical and subcortical regions, including the cingulate cortex, the hippocampus, temporal cortex, lateral hypothalamus, and amygdala. The orbitofrontal cortex is uniquely situated to facilitate communication between cortical and subcortical regions. It is involved in self-regulatory functioning, the social engagement and attachment systems, as well as the exploratory system. It has been proposed that early abuse, within the first two years of life, negatively impacts the maturation of the orbital prefronto-limbic system [19]. 

### Medial Prefrontal Cortex

The medial prefrontal cortex has been hypothesized to play a role in extinction of conditioned fear responses. By exerting control over certain limbic structures, such as the amygdala, the medial prefrontal cortex regulates the generalization of fear responses. In PTSD, positron emission tomography (PET) studies have shown a negative correlation between blood flow in the left prefrontal cortex and the amygdala. Additionally, medial prefrontal cortex dysfunction has been consistently observed in neuroimaging studies of PTSD. It has been hypothesized that this dysfunction is responsible for attentional and frontal deficits that can resemble dementia. This structure can also suppress the release of cortisol, a major stress hormone, through its action on the limbic HPA axis. Furthermore, medial prefrontal cortex may play a role in the retrieval of episodic memories, and therefore could contribute to the timeless nature of traumatic memories often observed in patients suffering from PTSD. The medial prefrontal cortex also may play a role in self-referential processing and thus may a play a significant role in the set of mindfulness skills that are often utilized to help mitigate the affect hyper-arousal often observed in those suffering from PTSD [70]. 

### Anterior Cingulate Cortex

Lanius and colleagues [69] noted that the anterior cingulate cortex “is a complex structure with multiple functions that have been shown to play a key role in the representation of subjective experience, integration of bodily responses with behavioral demands...and in emotional awareness”. Research in mammals has demonstrated that the anterior cingulate cortex has extensive connections with multiple brain structures, including the amygdala, hypothalamus, nucleus accumbens, ventral tegmental area, substantia nigra, raphe nucleus, locus coeruleus, periaqueductal grey area, and brainstem autonomic nuclei. Disruptions in the anterior cingulate cortex, as seen in PTSD, may provide a neural basis for affect dysregulation, including emotional hyper-arousal and numbing. Lanius *et al.* [69] further pointed out that the anterior cingulate cortex “...also plays significant roles in other responses crucial to preventing or surviving trauma, including pain, response selection, maternal behavior, vocalization [the separation cry/call?], and skeletomotor control”. Psychological trauma, including attachment trauma, in the first year of life has been observed to have a negative impact on the experience-dependent maturation of the circuits of the anterior cingulate cortex.

### Insula

The insula is a structure located in the cerebral cortex, thought to be involved in the emotional response to potentially distressing cognitive stimuli, interoceptive sensory stimuli, and somatic sensations. It may serve as an internal alarm system through its connection to the amygdala. Moreover, Lanius *et al.* [69] concluded: “As traumatized clients learn to slowly increase awareness of body sensations, movement, and impulses and to tolerate sensation and emotional arousal, changes in activation of the insula and medial prefrontal cortex may take place, thus increasing their ability for self-referential processing of bodily states and emotions. Clinically, they have observed that this ability to mindfully observe present-moment internal experience, in most instances, allows for down-regulation of defensive action systems and increased engagement of action systems related to daily life, especially the attachment, exploration, and sociability systems”.

### Trauma and Lateralization

Neuroimaging studies in PTSD suggest that there is increased brain metabolic activity in the right hemisphere during recall of traumatic memories, and decreased activity in the left hemisphere. PTSD related symptomatology seem to be associated with lateral differences, specifically right-hemispheric dominance. Psychological abuse has been demonstrated to be associated with an increased prevalence of left-sided EEG abnormalities and an increased prevalence of right-left hemispheric asymmetries [26].

There also seems to be a horizontal disconnect between lower and upper brain structures in PTSD as well as evidence demonstrating right-hemisphere dominance during traumatic memory recall. However, therapist and patient in treatment may have access to an alternative connection between the hemispheres referred to as the “subcortical bridge.” Despite the bifurcation of the hemispheres, this bridge remains undivided at lower brain centers. In general, it seems that language-based information cannot be exchanged readily across this bridge, whereas nonverbal information crosses more readily. Information indicating danger or safety can be transmitted across this subcortical bridge, leading to adaptive action, including the engagement of social support networks in order to ensure psychic and/or physical survival. A summary of the neurobiology of PTSD is seen in Fig. (**3**) and Table **2**. 

## VULNERABILITY AND RESILIENCE

Not all individuals who encounter stressful life events develop PTSD [71], and there is considerable interest in understanding what makes an individual vulnerable, and also what makes an individual resilient to the deleterious effects of traumatic events. Resilience and vulnerability are related concepts in a number of scientific disciplines but unlike resilience, vulnerability has not been conceptualized in a comprehensive manner. There are different disciplinary approaches to the concept of vulnerability. Vulnerability has been defined as the potential for loss [72] or the potential for casualty when exposed to a hazard or threat. The International Strategy for Disaster Reduction (2004) defines vulnerability as the predisposition of individuals or societies to be affected by disaster the inability to manage disaster [73]. They also divide vulnerability into external and internal factors. The external factors relate to external shocks and environmental stresses, whereas the internal factors are associated with inability to cope with trauma. Vulnerability is difficult to quantify but it may be increased or decreased depending on the actions taken in response to the trauma [74]. 

There is a plethora of research surrounding this issue focused on identifying mediating factors that are associated with increased (vulnerability) or decreased (resilience) risk for developing PTSD following traumatic stress [74] (see Table **1**). Many variables seem to be associated with the risk of developing PTSD. These include the nature of the precipitating event, genetic vulnerability, history of previous trauma, preexisting psychiatric disorder, family history of psychiatric disorder, insufficient support systems. The characteristics of the event (e.g., direct physical harm, proximity to threat, pattern duration and severity of the trauma), the characteristics of the traumatized individual, nature of the recovery environment, and the characteristics of family/social system (e.g., supportive, calm, nurturing *vs*. chaotic, distant, absent, anxious) all impact the risk of development of PTSD. Social support particularly *via *close meaningful relationships seems to be exceptionally important and it appears that positive social support of high quality can enhance resilience to stress, help protect against developing trauma-related psychopathology, decrease the functional consequences of trauma-induced disorders such as PTSD, and even reduce medical morbidity and mortality [75]. 

Despite strong evidence demonstrating the beneficial effects of family and social support on medical and psychological wellbeing, the field of psychiatry has contributed relatively little to developing, testing, and implementing effective evidence-based interventions aimed at increasing social support for patients and at-risk populations. Thus, impairments in personal adjustment, lack of supportive relationships, family history of PTSD, previous traumatic experiences and other existing mental disorders all play a role in vulnerability to developing PTSD. 

### Genetic Predisposition to PTSD: Vulnerability Risk

Based on twin studies it has been posited that genetic factors can influence the risk of developing PTSD. As in combat veterans, the development of PTSD symptoms after non-combat trauma also seems to be moderately heritable. Moreover, many of the same genes that influence exposure to assaultive trauma appear to influence susceptibility to PTSD symptoms in their wake.

The dopamine and opioid reward pathways of the brain are critical for survival by providing for the pleasure drives for eating, love and reproduction. These are called 'natural rewards' and involve the release of dopamine in the nucleus accumbens and frontal lobes. However, the same release of dopamine and production of sensations of pleasure can be produced by 'unnatural rewards' such as alcohol, cocaine, methamphetamine, heroin, nicotine, marijuana, and even compulsive behaviors such as gambling, eating, sex, and risk taking. Since only a minority of individuals become addicted to these compounds or behaviors, it is reasonable to ask what factors distinguish those who do become addicted from those who do not. It is no longer believed that the continuation of the destructive behaviors is entirely voluntary or that environmental factors play the major role. Since all of these behaviors have a significant genetic component, the presence of one or more variant genes presumably acts as a risk factor for these behaviors. Since the primary neurotransmitter of the reward pathway is dopamine, genes for dopamine synthesis, degradation, receptors, and transporters are reasonable candidates. Moreover, neurons containing receptors for serotonin, norepinephrine, GABA, opioid, and cannabinoid all seem to modify dopamine metabolism and dopamine neurons as well as drug treatment response.

Blum *et al*. [76] and Comings and Blum [77] have proposed that defects in various combinations of the genes for these neurotransmitters result in a Reward Deficiency Syndrome [76,78] and that such individuals are at risk due to behaviors used in seeking unnatural rewards. Because of its importance, the gene for the dopamine D2 receptor was a major candidate gene. Studies in the past decade have shown that in various subject groups the *Taq* I A1 allele of the DRD2 gene is associated with alcoholism, drug abuse, smoking, obesity, compulsive gambling, and several personality traits. One major area of importance is the potential for gene involvement in vulnerability to PTSD. A range of other dopamine, opioid, cannabinoid, norepinephrine, and related genes has since been added to the list. Like other behavioral disorders, these are polygenically inherited and each gene accounts for only a small per cent of the variance [77]. 

The first study on genes associated with vulnerability and PTSD came from the laboratory of Comings *et al*. [79]. In their study, subjects on an addiction treatment unit who had been exposed to severe combat conditions in Vietnam were screened for PTSD. Of 24 with PTSD, 58.3% carried the D2A1 allele. Of the remaining eight who did not meet PTSD criteria, 12.5% carried the D2A1 allele (*p* = 0.04). In a replication study of 13 with PTSD, 61.5% carried the D2A1 allele. Of the remaining 11 who did not meet criteria for PTSD, 0% carried the D2A1 allele (*p* = 0.002). For the combined group 59.5% of those with PTSD carried the D2A1 allele versus 5.3% of those who did not have PTSD (*p* = 0.0001). These results suggest that a DRD2 variant in linkage disequilibrium with the D2A1 allele confers an increased risk for PTSD, and the absence of the variant confers a relative resistance to PTSD. Along similar lines others have confirmed that the DRD2 A1 allele was associated with PTSD. However, this association was found only in the harmful drinkers. PTSD patients with the A1 (+) allele consumed more alcohol than patients with the A1 (-) allele. The importance of determining alcohol consumption in DRD2 association studies with PTSD is suggested [80]. These results suggest that negative association with the DRD2 A1 allele in PTSD may be due experimental flaws [81]. Further confirmation has revealed that the DRD2 A1 allele was responsible in part for an enhanced increase in social functioning following anti-depressant therapy compared to the DRD2 A2 allele [82]. More recently it was found that the 957C>T polymorphism in the DRD2 gene is one of the genetic factors for susceptibility to PTSD [37].

Other dopaminergic genes may also contribute to PTSD vulnerability. Specifically, Segman *et al*. [83] evaluated 102 chronic PTSD patients and 104 carefully-documented trauma survivors who did not develop PTSD. Significant excess of nine repeat allele was observed among PTSD patients (43% *vs*. 30.5% in trauma survivor controls; chi(2) = 6.3, df = 1, *p* = 0.012). An excess of nine repeat homozygous genotype was also observed in PTSD (20.43% in PTSD *vs.* 9.47% in trauma survivor controls; chi(2) = 6.11, df = 2, *p* < 0.05). These findings suggest that genetically determined changes in dopaminergic reactivity may contribute to the occurrence of PTSD among trauma survivors. Others have also associated MAO-B activity, and genotyping of this gene might be used as a peripheral marker of the psychotic symptoms in PTSD [84]. 

In terms of vulnerability genes associated with PTSD risk Mustapic *et al*. [85] evaluated DBH-1021C/T gene polymorphism in combat veterans and found with (N = 133) or without (N = 34) in veterans with chronic PTSD. Similar frequencies in genotype or allele distribution were found between veterans with or without PTSD. War veterans with PTSD had lower dopamine beta hydroxinase (DBH) activity, associated with the DBH-1021C/T variant in DBH genes, than veterans without PTSD. A significantly lower plasma DBH activity was found in combat veterans with PTSD carrying the CC genotype as compared to veterans without PTSD carrying the corresponding genotype. Since both groups were exposed to the same trauma, it is possible that a preexisting trait difference in regulation of nor-epinephrine function contributed to a differential vulnerability to develop PTSD, or that the regulation of DBH expression was different in response to trauma. The results suggest that that genotype-controlled measurement of plasma DBH activity might be used as a potential biological marker of the response to trauma, and that further studies of DBH and other loci related to dopamine and nor-adrenolin in PTSD are warranted.

Lee *et al*. [86] examined the possible association between the serotonin-transporter-linked polymorphic region (SERTPR) and PTSD. The genotype and allele frequencies of the SERTPR were analyzed in 100 PTSD patients and 197 unrelated healthy controls using a case-control design. The frequency of the s/s genotype was significantly higher in PTSD patients than in normal controls. These findings suggest that the SERTPR s/s genotype is one of the genetic factors for the susceptibility to PTSD.

Moreover, acute stress, which can also induce PTSD in at risk individuals, has been found to alter the expression of cholinergic genes. In fact results suggest a model in which robust cholinergic stimulation triggers rapid induction of the gene encoding the transcription factor c-Fos. This protein then mediates selective regulatory effects on the long-lasting activities of genes involved in acetylcholine metabolism [7].

GABA systems have been implicated in the pathogenesis of anxiety, depression and insomnia. These symptoms are part of the core and comorbid psychiatric disturbances in PTSD. In a sample of Caucasian male PTSD patients, dinucleotide repeat polymorphisms of the GABA(A) receptor beta 3 subunit gene were compared to scores on the General Health Questionnaire-28. A study by Freusner *et al. *[87] indicates that in a population of PTSD patients, heterozygosity of the GABRB3 major (G1) allele confers higher levels of somatic symptoms, anxiety/insomnia, social dysfunction and depression than found in homozygosity. 

Additionally, previous studies have reported associations between apolipoprotein E (APOE) genotype, cognitive function, and psychopathology in psychiatric populations. Freeman *et al.* [88] investigated the associations between APOE allele status, memory function, and symptom severity in PTSD subjects. The presence of the APOE 2 allele was associated with significantly worse re-experiencing symptoms and impaired memory function in this population. Furthermore, studies have also shown that a putative association of the cannabinoid receptor (CNR)1 gene may be a risk factor for attention deficit hyperactivity disorder (ADHD), and possibly PTSD [89]. 

In terms of prolonged memory of traumatic experiences, recent studies in mice have been suggestive that Myo6 gene may be selectively and rapidly up-regulated to play a hitherto unidentified role in the maintenance of the integrity and functionality of the hippocampus after traumatic stress [90]. 

Using microarray assays others have associated PTSD with decreased activity in the dorsolateral prefrontal cortex, the brain region that regulates working memory and preparation and selection of fear responses. Su *et al*. [91] investigated gene expression profiles in dorsolateral prefrontal cortex (Brodmann’s area 46) of postmortem patients with (n=6) and without PTSD (n=6) using human mitochondria-focused cDNA microarrays. Their study revealed PTSD-specific expression fingerprints of 800 informative mitochondria-focused genes across all of these 12 samples from Brodmann’s area 46, and 119 (+/->1.25, p<0.05) and 42 (+/->1.60, *p*<0.05) dysregulated genes between the PTSD and control samples. Quantitative RT-PCR validated the microarray results. These fingerprints can essentially distinguish the PTSD dorsolateral prefrontal cortex from controls. Of the 119 dysregulated genes (+/-> or =125%, *p*<0.05), the highest percentages were associated with mitochondrial dysfunction (4.8%, *p*=6.61 x 10(-6)), oxidative phosphorylation (3.8%, p=9.04 x 10(-4)), cell survival-apoptosis (25.2%, *p*<0.05) and neurological diseases (23.5%, *p*<0.05). Fifty dysregulated genes were present in the molecular networks that are known to be involved in neuronal function-survival and contain seven targets for neuropsychiatric drugs. Thirty of the dysregulated genes are associated with a number of neuropsychiatric disorders. Their results indicate mitochondrial dysfunction in the PTSD dorsolateral prefrontal cortex, and provide the expression fingerprints that may ultimately serve as biomarkers for PTSD diagnosis and the drugs and molecular targets that may prove useful for development of remedies for prevention and treatment of PTSD.

Moreover, it has been found that several single nucleotide polymorphisms (SNPs) in FK506 binding protein 5 (FKBP5) interact with childhood trauma to predict severity of adult PTSD [92]. These findings suggest that individuals with these SNPs who are abused as children are more susceptible to PTSD as adults. This is particularly interesting given that FKBP5 SNPs have previously been associated with peritraumatic dissociation (that is, dissociation at the time of the trauma) [93] which has itself been shown to be predictive of PTSD [6, 94]. Furthermore, FKBP5 may be less expressed in those with current PTSD [93-95]. There is a new finding in which two neurotransmitters are reduced after traumatic brain injury: hypocretin (orexin) and melanin concentrating hormone neurons (MCH) in the hypothalamus [96]. 

In summary, a number of genes have been associated with PTSD. These include serotonergic, dopamineergic (DRD2, DAT, DBH), glucocorticoid, GABA (GABRB), and apolipoprotein systems (APOE2), brain-derived neurotrophic factor, Monamine B activity, CNR1, Myo6, CRF-1 and CRF-2 receptors, neuropeptide Y (NPY), orexin and MCH. However, the complex etiology of PTSD, for which experiencing a traumatic event forms a necessary condition, makes it difficult to identify specific genes that substantially contribute to the disorder. Gene-finding strategies are difficult to apply. Interactions between different genes and between them and the environment probably make certain people vulnerable to developing PTSD [28, 92, 94-96]. More gene-environmental studies are needed that focus more narrowly on specific, distinct endophenotypes and on influences from environmental factors.

### Resilience

Masten [97] refers to resilience as a pattern of positive adaptation in the context of significant risk or adversity. It is the process to “bouncing back” from difficult experiences. Janoff and Bulman [98] also described traumatic events as rebuilding shattered assumptions; traumatic life events shatter our fundamental assumptions about our world and ourselves: the benevolence of the world; the worthiness of the self; and the world as meaningful. In the aftermath of these extreme experiences, coping involves the task of reconstructing an integrated assumptive world that incorporates the traumatic experience. Resilience is different from recovery in that it represents a distinct and empirically separable outcome trajectory from that normally associated with recovery from trauma.

Bonanno [74] has defined resilience as the ability to maintain a state of normal equilibrium in the face of extremely unfavorable circumstances. To enhance resilience, it is necessary to have an understanding of its determinants. Various factors such as beliefs, attitudes, coping strategies, behaviors and psychosocial cohesion have been suggested as conveying protection or endorsing resilience in the face of trauma. Other factors, such as religious faith and socio-political effectiveness, have been argued to produce resilience because they induce a sense of self control. Each mediating factor can be related to the degree to which they either prolong or attenuate and individual’s stress-response activation resulting from the traumatic experience [98]. Resilient individuals may show insight, have initiative, use humor and be creative and independent. It is a dynamic quality and there may be discontinuities in resilience as people, situations, opportunities, and environment change.

Psychological resilience is seen as a relatively stable personality trait characterized by the ability to bounce back from negative traumatic events and to return to a state of harmony and balance. Flexible adaptation to the ever-changing demands of life builds resilience. Resilient people are optimistic, they maintain hope about future outcomes. Such optimism is associated with the use of active, problem-focused coping when dealing with stressful life events [100].

In fact many resilient people have optimism, even in dire situations. While in some people optimism appears to be genetic, it can also be learned. For example, cognitive-behavioral therapy in part is designed to enable people to view their situation in a more positive light and to see ways out of a difficult situation [101]. Having a moral compass or a set of beliefs that few things can shatter can get a person through very tough times. Indeed, faith or spirituality has some overlap with a moral compass, and for some people can provide comfort and a sense of optimism and hopefulness in the face of traumatic situations [100]. 

Resilient people are self-efficient; they believe that they have the skills necessary to effectively cope, manage or withstand hardship and to accomplish any difficult task. Lack of predictability and control are the central issues for the development and maintenance of PTSD. Resilient people have a *sense of mastery*, they believe that they can exert positive control over the environment. They believe that by breaking down complex problems into smaller, more manageable tasks and goals, they can achieve a series of immediate successes [101]. These successes enhance their feeling of mastery and control over the problem [101]. Another characteristic that resilient people have is cognitive flexibility, which enables the person to see the experience as an opportunity for personal growth. Meaning that the experience of severe trauma can be used to discover and develop new skills and strengths, and gain insights. What is especially interesting about this is that it seems to imply that PTSD is not caused only by the negative events themselves, but by how individuals interpret those events [102].

Recently Pitman’s group concluded from a series of experiments that found combat veterans with PTSD have functional abnormalities in the anterior cingulate cortex, amygdala, and hippocampus. They most recently found, enhanced resting metabolic activity in the dorsal anterior cingulate cortex/midcingulate cortex (dACC/MCC). This appears to represent a familial risk factor for developing PTSD after exposure to psychological trauma [103]. More- over, a clinical characteristic of PTSD is persistently elevated fear responses to stimuli associated with the traumatic event. Milad *et al.* [22] found that fear extinction is impaired in PTSD. They further suggest that dysfunctional activation in brain structures that mediate fear extinction learning, and especially its recall, underlie this impairment in combat veterans. 

The question of whether or not there is a genetic or an inborn acquired biological marker for PTSD is best exemplified by the recent work of Metzger [104]. These investigators explored an identical-twin, case-control design to investigate whether these abnormalities reflect pre-trauma vulnerability or the acquired consequence of PTSD. Vietnam combat veterans and their non-combat-exposed, identical twins completed a three-tone oddball task. It was found that Veterans with PTSD had delayed target N2 latencies compared to veterans without PTSD. In a small non-medicated, nonsmoking subsample, veterans with PTSD also had significantly diminished target P3b amplitudes. A mixed-model, random-effects analysis on the non-medicated, nonsmoking subsample that included the combat-unexposed co-twins showed a significant Diagnosis x Combat Exposure interaction for target P3b amplitude. Results replicate increased N2 latency and diminished P3b amplitude in PTSD and suggest that diminished P3b amplitude is an acquired condition in PTSD.

Through the era of protinomics we are finding gene expressions that are impacted by our environment, and stress is a powerful environmental pressure that, because of gene polymorphisms, predicts clinical outcomes in terms of pessimism and optimism. This fact leads us to believe that genetic diagnosis and genome targeting of treatment of PTSD will ultimately result in a more personalized approach to this complex devastating disorder. Based on the available literature and studies it seems prudent that soldiers should be subject to genetic, neuroimaging and psychometric testing prior to combat. This screening for vulnerability to PTSD could reduce the enormous burden placed on society, the government and the famlies of its victims. 

## CLINICAL ISSUES: FROM BENCH TO BEDSIDE

A considerable amount of research has found that trauma has negative impacts on different body systems, impacting physical, psychological, and behavioral health. In other words, trauma may lead to poor health outcomes. Research also shows that these neurochemical changes may relate to abnormalities in thyroid and other hormone functions, and to increased susceptibility to infections and immunologic disorders associated with PTSD. The psychological and behavioral effects of PTSD on health may be accounted for in part by comorbid depressive and anxiety disorders. There is some evidence that neurochemical changes in PTSD are related to cardiovascular, gastrointestinal, and musculoskeletal disorders. These studies found that self-report of circulatory disorders and symptoms of cardiovascular trouble were associated with PTSD in veteran populations, civilian men and women, and male firefighters [6, 20, 105, 106,]. Among studies that have examined PTSD in relation to cardiovascular illness *via *physician diagnosis or laboratory findings, PTSD has been consistently associated with a greater likelihood of cardiovascular morbidity. In a recent study, researchers used electrocardiogram (ECG) findings to compare the cardiovascular function of Vietnam veterans with PTSD to the cardiovascular function of veterans without PTSD. After controlling for risk factors such as alcohol consumption, weight, current substance abuse, and smoking, in addition to controlling for current medication use, PTSD was found to be associated with nonspecific electrocardiogram abnormalities, atrioventricular conduction defects, and infarctions. Because the PTSD group in this study included only those veterans with severe PTSD, it is important to interpret this study with caution. It is unknown whether men with less severe PTSD would show the same electrocardiogram abnormalities [20]. There have been only a small number of studies specifically addressing cardiovascular abnormalities in women [6]. Gastrointestinal and musculoskeletal systems dysfunctions have also been shown to be associated with PTSD, but the relationship of PTSD to these two systems has not been as extensively researched as the relationship between PTSD and the cardiovascular system [105]. The majority of the studies that have been conducted have gathered information about veterans, but a study of civilian young men and women found that there is a relationship between gastrointestinal symptoms and PTSD. Similarly, researchers found that PTSD was related to musculoskeletal symptoms among male firefighters [106]. Additional research is needed to learn more about how these and other bodily system troubles may be related to PTSD. 

### Case Reports 

The following three case reports from the Department of Psychiatry, University of Florida, College of Medicine, Gainesville, Florida reflect the unfortunate circumstances that many patients with PTSD must deal with and potentially resolve.

#### Rape Victim 

Maureen is a 24 year old obese female who presented to our facility after a 9 day drug detoxification followed by 1 month of primary drug rehabilitation. She was referred to our facility due to concern that she was a victim of sexual abuse and suffered from co-exisisting PTSD. It was felt that this was hindering her from maintaining sustained recovery from polysubstance addiction with a preference for IV opioids: alprazolam, and crack cocaine. She had been in multiple rehabilitation facilities since the age of 15 and had been unable to achieve abstinence longer than two months. We were advised that she was a “runner”, having absconded from the referring facility only to return intoxicated, bruised, beaten and raped. There were also reports of her wandering off while on campus, and being found “spaced out “with no recollection of how she ended up at her destination”. She also had a history of self mutilation (cutting), one intentional drug overdose, running away from home and living on the streets as well as prostitution. She had also a history of violent short term unstable relationships. The referring facility sent her with the diagnosis of PTSD, depression, dissociative disorder, polysubstance dependence and borderline personality disorder.

Maureen was a single child from a well to do family. She reports sketchy “dream like” memories of her father abusing and raping her over a 10 year period between the ages of 6 and 16 when she ran away from home. Her father, a minister claimed she was making this up and that she was violent and unstable. He was willing to pay for her treatment. Her mother also denied that there was any validity to these claims. There was never any objective evidence found that corroborated these claims. Though her father willingly paid for her treatment, he remained distant, unavailable for phone conferences and unwilling to participate in any family sessions. He did not visit her nor call during her nine month stay.

Upon arrival she underwent psychiatric evaluation. Medications upon arrival included escitalopram and PRN quetiapine for anxiety. After evaluation, quetiapine was discontinued due to her problem with weight control as well as the perception that she was abusing it for its sedative effect. Ziprasidone was added and titrated up to 80 mg bid, and prazosin QHS was prescribed for violent dreams. It was noted that she appeared sedated upon arrival. A urine drug screen was done which was positive for benzodiazipines. When confronted, she initially denied this, but a thorough search of her belongings revealed 1mg clonazepam tablets concealed in her alarm clock. She admitted to taking clonazepam during the entirety of her stay at the primary facility, and it was confirmed that their UDS would not pick up this particular benzodiazepine. She underwent a clonazepam taper at our facility without any problems.

During a three week primary phase she was introduced to relaxation and self soothing exercises, and developed a trusting relationship with her therapist. She then began the second phase of treatment. While constructing a time line of her life, there were significant problems with remembering time periods and events. Her roommates were suspicious that she had resumed cutting herself. Examination of her medial upper arm revealed numerous lacerations and a search of her cabin revealed razor blades. She explained that she had been cutting on and off for many years, had stopped it for awhile but resumed it when she began doing her assignments because it “made her feel calm”. Her treatment stay was also complicated by frequent visits to the medical unit for headaches and stomach aches. This intensified with phase 2 of treatment. There were also intense outbursts when the medical staff refused to grant her sick passes or change her medication to include daytime doses of quetiapine. There were also dissociative episodes where she would wander off during group without any recollection of doing so. She was placed back in phase 1 for several weeks then successfully transitioned to phase two. She complained of a constant state of anxiety and feelings that her father was spying on her. She also began suffering violent dreams and bouts of screaming and crying during groups when relating her experiences. Her memories were vivid during these episodes, claiming that she could feel his presence and smell his aftershave. She also had severe drug cravings and was found attempting to sneak away while attending off campus Narcotics Anonymous meetings.

Progress was slow as it was felt that a gradual and delicate approach to exposure therapy was needed to avoid retraumatization. Finally, after many months she was able to complete her timeline and write a letter to her abuser though she refused to mail it. She also began to embrace NA, obtained a sponsor, and began working the 12 steps. Though her anxiety remained at a fairly high level, her depression improved and her dissociative episodes decreased. Finally, after nine months she entered a half way house and began to do volunteer work at a rape support group. She continues to participate in outpatient therapy. She still has occasional flashbacks, though has been unable to identify triggers. She feels very uneasy in the proximity of churches stating that the remind her of her father. She is also unwilling to attend any AA or NA meetings that are held in churches for the same reason. She is continuing to work with a therapist on these issues. 

#### Death Threat Victim

Doug is a 44 year old male who presented to our pain medicine clinic with multiple pain complaints including groin, abdominal, knee, back, chronic headaches and “all over” pain for which he carried the diagnosis of fibromyalgia. This was his second appointment as he had missed his first due to “transportation problems”. During the history, he seemed very anxious and hyper-alert. It was also noted that he had moved the chair to the far wall facing the door. Doug had multiple complaints of difficulty sleeping, irritable bowel syndrome, anxiety and various pain disorders. He had a right rotator cuff injury and multiple urological evaluations for testicular pain where no etiology was found. He had had a non-chaotic childhood. He was an only child with an over attentive mother. He had attended college and had worked for a well known industrial corporation receiving a fairly good salary until four years ago. Currently he is unemployed and living alone off of savings and a small stipend provided by his parents. He had no car.

There were some inconsistencies during his history related to the onset of the various pain complaints, but he kept alluding to a “work injury” approximately four years ago, though he would not initially provide specifics. Up until approximately an hour into the interview, he was being evasive in providing anything specific. Doug then became tearful. After some reassurance and coaxing he explained that his problems began at work approximately four or five years ago. He also denied ever sharing this with anybody else due to fear and shame. He reported being very meek by nature and had been picked on all of his life. While at work a supervisor tormented him relentlessly and ridiculed him in front of his peers. Over time the supervisor became increasingly abusive with “accidental” pushing and shoving as well as grabbing is genital area as well as threats of perverse sexual molestation. He was also told that if he told anyone or complained, that the supervisor, who frequently bragged about his violent history as well as his gun and knife collection would “hunt him down and kill him”.

As this continued over many months, Doug began to call in sick frequently to work. He began having difficulty sleeping and bouts of diarrhea. He finally “summed up his courage” and went to one of the managers and complained that he was being verbally abused by his supervisor and asked to be relocated. The supervisor was interviewed, denied any abuse and agreed to be a less forceful boss with Doug. A meeting was set up between the three where the supervisor apologized to Doug. Things got better at work with both Doug and the supervisor avoiding each other. Several weeks later, the supervisor came up behind Doug grabbed his testicles and began squeezing saying that he was indeed going to kill him. According to Doug, he briefly passed out from the pain. He then left work, noted that his testicles were bruised and swollen, went to the emergency room and related falling while straddling a bicycle, injuring himself. He was given pain pills and set up with urology, though did not follow through with the appointment. He went back to the manager, stated he had been “grabbed “by his supervisor, again requesting relocation. The manager said he would have a talk with the supervisor and would think about the transfer. After a few days, the supervisor brushed by him and said “I am going to kill you”. Doug never went back to work. He moved to a different state and found a small apartment.

Since that time he has become increasingly reclusive. He denies having any friends or social life. He rarely leaves his apartment. He sold his car to a brother and now walks about everywhere. He has purchased several guns. He keeps one his pillow at night, and the others scattered throughout his apartment. He claimed to have one in his backpack. He believes his ex-supervisor is stalking him. He has heard that the supervisor left his job a couple of months after Doug did. He cannot sleep at night, has violent dreams where he is abducted, tortured and killed by his ex-supervisor or his friends. He is hyper alert and “jumps at the slightest noise”. He admitted missing his first appointment because he was so fearful of public transportation.

He was placed on an SSRI, NSAIDS, received knee injections which have been helpful. He is being evaluated by urology for testicular pain, though no etiology has yet been found. He was referred to psychiatry for depression and psychotropic medical management. He is also being seen by a psychologist where he is receiving outpatient counseling for PTSD. He has purchased a used car, and is looking into disability. He has also shared his story with his brother and others and has sought out the services of an attorney who is preparing a case against the corporation. His many somatic complaints are slowly improving. 

#### Iraq Veteran

Jim is a 32 year old veteran of the war in Iraq who presented to our facility with the diagnosis of PTSD, depression and alcoholism. While in Iraq, he and a close friend encountered an improvised explosive device while driving together. The explosion resulted in the driver sustaining critical craniofacial injuries while Jim sustained a broken femur. Jim was trapped in the passenger seat and was able to hold his injured friend’s airway open until the medics arrived. Unfortunately, his friend, though initially conscious and lucid, died as they were being extricated from the damaged vehicle. Jim underwent successful emergency ORIF (Open Reduction Internal Fixation) of his femur, which was broken in the explosion and recovered full use. Jim returned to the states and was given a hero’s welcome by his home town. According to his wife, he was uncharacteristically quiet during the drive home. He became increasingly withdrawn and experienced crying spells. Though previously known for his ability to solve problems and multitask, he was overwhelmed by even the smallest problems. His mood was labile, his temper short and he smoked heavily. Two weeks later, he and his wife had a small disagreement and he slapped her, and immediately apologized and began to cry. He would toss and turn in his sleep, crying out at times. His dreams were vivid, and violent, and frequently the theme was observing one of his friends or a family member in a perilous situation while he was physically paralyzed, unable to speak or move and forced to helplessly watch them die. Though always a heavy drinker and a gregarious outgoing entertainer, he now avoided his friends and began drinking even more heavily, while alone. He would drink to intoxication every night and incurred a DUI when he was noted to be swerving. His 3 year old daughter was in the car at the time. He was always on edge and would startle when the phone or doorbell rang. He was extremely lethargic during the day, his speech and movement increasingly slow. He admitted to feeling guilty about surviving the explosion, and would have episodes that resembled panic attacks where he believed that he was back in the destroyed car with his dying friend and would begin screaming for help. He also avoided watching the news and refused to talk about Iraq. He would frequently drive around town alone at night, drinking alcohol and talking to himself. After his second DUI, he was sent to our rehabilitation facility that specialized in alcoholism, drug addiction and PTSD. While in treatment, Jim was introduced to 12 step recovery, which he embraced, obtaining a sponsor who was also a veteran that had sustained war injuries. He was in treatment for three months where he learned techniques to deal with anxiety and obtained treatment for depression. He attended counseling groups with other veterans, and over time became increasingly able to share his experiences without reliving them. He wrote letters to his dead friend that he read aloud in group. He was also very adept at helping others who had had similar experiences, and developed a career plan to one day be a counselor. His violent dreams faded with time, his relationship with his wife improved and he devoted himself to an exercise and nutrition regimen, quit smoking and began training with the goal of running the Marine Core Marathon in his friends honor.

## NEW DIAGNOSTIC OPPORTUNITIES

As discussed in this review, traumatic experiences can produce PTSD, a very debilitating condition. Unfortunately no biomarker except for the promise of genotyping for a validated PTSD gene panel to determine high risk [107] existed until most recently when Georgopoulos *et al. *[108] identified the synchronous neural interactions (SNI) test. SIN assesses the functional interactions among neural populations derived from magneticencephalographic (MEG) recordings and can successfully differentiate PTSD patients from healthy control subjects. These new findings document robust differences in brain function between the PTSD and healthy controls. While we ponder the potential of pre-existing genetic antecedents this work underscores differential diagnosis which possess the potential for screening and possibly monitoring disease progression and the effects of therapy. It is to be noted that one of us (ERB) has suggested that since the MEG is very expensive it should be appropriate to diagnose PTSD and brain function with Brain Electro-Activity Mapping (BEAM) in clinical primary care settings. It may be equally important to genotype PTSD patients for a number of neurotransmitter genes in conjunction with the BEAM test and or SNI.

### Treatment Goals

The prognosis for successful treatment of PTSD includes several factors beginning with the length of time that has passed since the trauma, as early intervention is effective in reducing symptoms and helping to prevent secondary chronic morbidity. The sufferer attitude to treatment, their capacity to tolerate distress, the availability of support and the extent of comorbidity are important factors. 

The five main treatment goals are reducing the core symptoms, improving stress resilience, improving the quality of life, reducing disability and reducing comorbidity. Cognitive behavioral therapy programs, exposure therapy and anxiety management are all employed, with the strongest evidence of success in exposure therapy. Anxiety management techniques show good results after rape [109]. Alternative medicine is showing promise in the treatment of PTSD. Stress management techniques such as the deep relaxation response achieved through breathing techniques could be useful in controlling and/or responding to triggered alarm responses brought on by the recurrent memories. Aerobic exercise improves stamina, strength and general wellbeing and reduces aggressiveness. A study involving a group of PTSD veterans who practiced yoga exercises and meditation several times each day over six weeks produced a reduction in all symptoms and a general improvement in health and enthusiasm [110]. 

Other approaches might target the immune system since immunity is one of the areas directly affected in PTSD. Circulating epinephrine and CRF are synergistic and act with cortisol to inhibit the function of the immune system [111]. 

### Enumerated Treatment Opportunities

In recent years the biologic origins of PTSD have been extensively researched and there are basic conceptual requirements that need to be addressed in a clinically acceptable and scientifically sound paradigm shift in treating PTSD. Based on our current knowledge there are many traditional and non-traditional treatment opportunities these include:

- Intervene early to reduce vulnerability by using genetic screening for and treatment with neuronutrient therapy both post disaster and abuse trauma and prior to military combat.
- Support resilience by the implementing interventions that result in strong social support for victims of disaster, war and abuse and by using cognitive behavioral therapy to develop; cognitive flexibility and a sense of mastery.
- Treat the symptoms of PTSD with cognitive therapy, behavioral techniques, stress reduction techniques and alternative therapies to reduce stress, reinstate self-referral and integrate the traumatic event into the victim’s life. Use pharmacological and/or neuronutrient therapy for neurological symptoms of PTSD and comorbid psychiatric illness including substances abuse disorder, anxiety, depression and psychosis.
- Treat reversible changes to the neuroanatomy of the brain pharmacologically and with brain training therapies.
- Early and vigorous treatment for physiological sequelae of chronic stress to reduce suffering, disability and cost.

## FUTURE PERSPECTIVE: UNDERSTANDING PTSD

PTSD may promote poor health through a complex interaction between biological and psychological mechanisms. Current thinking is that the experience of trauma brings about neurochemical changes in the brain. These changes may have biological, as well as psychological and behavioral, effects on one's health. For example, these neurochemical changes may create a vulnerability to hypertension and atherosclerosis heart disease that could explain in part the association with cardiovascular disorders. Research also shows that these neurochemical changes may relate to abnormalities in thyroid and other hormone functions, and to increased susceptibility to infections and immunologic disorders associated with PTSD. The psychological and behavioral effects of PTSD on health may be accounted for in part by co-morbid depressive and anxiety disorders. Many people with PTSD also experience depressive disorders or other disorders. Depressed individuals report a greater number of physical symptoms and use more medical treatment than do individuals who are not depressed. Depression also has been linked to cardiovascular disease in previously healthy populations and to additional illness and mortality among patients with serious medical illness [112]. Hostility, or anger, is another possible mediator of the relationship between PTSD and physical health [25]. It is commonly associated with PTSD and decades of research on the health risks associated with the Type A behavior pattern have isolated hostility as a crucial factor in cardiovascular disease. A number of studies have found an association between PTSD and poor cardiovascular health. Among studies that have examined PTSD in relation to cardiovascular illness *via *self reporting of circulatory disorders, physician diagnosis or laboratory findings, PTSD has been consistently associated with a greater likelihood of cardiovascular morbidity. PTSD and poor health also may be mediated in part by behavioral risk factors for disease such as smoking, substance abuse, diet, and lack of exercise.

### Proposal

It is our proposal that in order to successfully treat PTSD, each patient should be genetically tested prior to treatment and then by virtue of certain polymorphisms, target these resultant genetic deficiencies with compatable substances (i.e pharmacologic and or nutrients)-“body friendly” [16]. 

## HYPOTHESIS

Because genes contribute both to vulnerability and to resilience, we propose that successful treatment of PTSD will benefit from genetic testing prior to treatment. When certain polymorphisms are found, clinicians can target these resultant genetic deficiencies with compatible substances (i.e., pharmacological compounds and/or body friendly nutrients) [16]. In this regard, tests for the following genes are recommended: serotoninergic, dopaminergic (DRD2, DAT,DBH), glucocorticoid, GABAergic (GABRB), apolipoprotein systems (APOE2), brain-derived neurotrophic factor, Monamine B, CNR1, Myo6, CRF-1 and CRF-2 receptors, and neuropeptide Y (NPY), Orexin and MCH. Treatment in part should be developed that would up-regulate expression of these genes to bring about a feeling of well being, as well as a diminished recall of the traumatic event. 

Early diagnosis is especially important. Thus, for combat related PTSD, we suggest that new military recruits be offered the opportunity to be genetically tested for a predisposition (vulnerability) to PTSD if it is likely that they will be exposed to multiple traumatic events. Understanding the extent of vulnerability through genetic testing prior to entry into the military might also prevent domestic violence by retuned PTSD soldiers following discharge [113]. The Army has declared soldiers' mental health a top priority [113], and it is our belief that screening soldiers for vulnerability to PTSD can decrease societal cost and individual victimization.

## SUMMARY AND CONCLUSIONS

Trauma the stressor stimulates SNS *via* neurochemical messengers that include neurotransmitters and hormones. Genetic differences alter the impact of SNS stimulation on gene expression and results in resilience or vulnerability of individuals to PTSD. Particularly in vulnerable individuals the neurochemical response to stress, including alterations in the function of the neurotransmitters involved in both the response and connectivity of the mesolimbic system and the magnitude of the catecholamine response, results in the three categories of symptoms of PTSD; re-experiencing, avoidance and increased arousal. The duration and extent of the cortisol response, causes alterations in neurophysiology that also support PTSD symptomatology. Untreated the symptoms of PTSD become a secondary stressor leading to the development of comorbidities. These include psychiatric disorders like depression and anxiety and the physiological symptoms of chronic stress including hypertension, heart disease, diabetes, gastrointestinal and musculoskeletal disorders. In an attempt to ameliorate PTSD symptoms, sufferers become involved in substance abuse and other behaviors associated with Reward Deficiency Syndrome [76, 77], leading to social dysfunction for example homelessness among veterans, family breakup, loss of productivity and criminality. Additionally, as a result of the psychotic symptoms (for example, flashbacks, hyper-vigilance and paranoia) victims of PTSD become perpetrators of domestic violence.

Although the biological underpinnings of human response to trauma are extremely complex, there are many points between the trauma and the development of PTSD symptomatology and comorbidities where interventions can be made to reduce morbidity and mortality. From this comprehensive review of the antecedents of PTSD, many areas for treatment with accepted modalities have been elucidated. Many more areas ripe for translational research have been identified. The understanding of the gene-environmental interactions will provide a diagnosis and treatment map to illuminate one’s vulnerability and or resilience for PTSD. We are suggesting that with new health legislation in USA that requires evidence based treatment, the wars in Afghanistan and Iraq, the global ecological crisis, and natural disasters, PTSD is a public health issue in need of urgent attention. 

## Figures and Tables

- Abnormalities in the sympathetic branch
- Severe stress causes dysregulation of locus coeruleus causing hypersensitivity to external stimuli
- Studies of combat veterans *vs.* non-combat veterans
- Differences in combat veterans
- Increased heart rate and anxiety during combat-related stimuli
- Increased urinary excretion of norepinephrine in PTSD patients
- Abnormalities of the Hypothalamic Pituitary Adrenal Axis (HPA)
- In acute/chronic stress and major depression:
- Cortisol levels are increased
- In PTSD, sensitization of HPA occurs
- Cortisol levels are chronically decreased
- Patients with PTSD show lower cortisol levels up to 50 years after initial trauma
- Lower cortisol levels immediately following trauma can be a risk factor for developing PTSD at a later date
- Hippocampus
- MRI measurements have shown it to be smaller in patients with PTSD
- Correlates with severity of traumatic exposure, cognitive deficits, and PTSD symptoms
- Functional neuroimaging while stimulating with traumatic material has shown:
- Hyperactive amygdala
- Anterior paralimbic regions are activated
- Decreased activity in anterior cingulate cortex
- Decreased blood flow to the hippocampus
- All this goes to show that in the model of PTSD
- Amygdala and related structures are overactive
- Also known as “ hot and emotional” memory system
- Hippocampus and anterior cingulate cortex is underactive
- Also known as “cool and cognitive” memory system

- Highly resilient operations soldiers tend to have high level of NPY.
- In contrast, Veterans diagnosed with PTSD have low level of NPY.
- Negative correlation has been found between DHEA and PTSD symptoms.
- Positive correlation has been found between DHEA/Cortisol ratio and performance among Special Forces soldiers during high stress training.
- DHEA + Allopregnalolone = Resilience to stress by helping terminate HPA activation and preventing harmful effect of glucocorticoids.

**Table 1:** The Neurochemical Response Patterns to Acute Stress

Neurochemical	Acute Effects	Brain Regions	Key Functional Interactions	Association with Resilience	Association with Psychopathology
Cortisol	Mobilized energy, arousal, attention and fear.	Prefrontal cortex, hippocampus, amygdala	Increases amygdala & Hypothalamus corticotropin-releasing hormone (CRH).	Stress-induced increase negative feedback	hypercortisolemia-depression, hypertension,
Dehydroepian-drosterone (DHEA)	Has positive mood effects	hypothalamus	Antiglucocorticoid actions	High DHEA-cortisol ratios have effects regarding PTSD and depression	Low DHEA cause PTSD and depression
CRH	Fear behaviors, increased motor activity, reduced reward expectations	Prefrontal cortex, cingulate cortex, mid brain structures.	CRH-1 anxiolytic, increases cortisol and DHEA.	Reduced CRH release.	increased CRH may predispose to PTSD and major depression
Locus coeruleus-norepinephrine system	General alarm function increased arousal, increased attention, fear memory formation.	Prefrontal cortex, amygdala, hippocampus, hypothalamus	Activates sympathetic axis, inhibits parasympathetic, stimulates hypothalamic CRH	Reduced responsiveness of locus coeruleus-norepinephrine	locus coeruleus-norepinephrine system leads to chronic anxiety, hypervigilance.
Neuropeptide Y	Anxiolytic; counteracts CRH and the locus coeruleus-norepinephrine	Amygdala, hippocampus, hypothalamus, locus coeruleus	Reduces CRH-related actions at amygdala, locus coeruleus	Adaptive increase in amygdala neuropeptide Y	Low neuropeptide Y response to stress by increased PTSD and depression
Galanin	Anxiolytic; counteracts with norepinephrine system; impairs fear conditioning	Prefrontal cortex, amygdala, hippocampus, hypothalamus, locus coeruleus	Reduces the anxiogenic effects of norepinephrine	increase in galanin is associated with reduced stress, anxiety and depression	low galanin response to stress is associated with increased PTSD and depression
Dopamine	low nucleus accumbens dopamine associated with helpless behaviors	Prefrontal cortex, nucleus accumbens, amygdala	Reciprocal interactions between cortical and sub-cortical dopamine systems	Cortical, subcortical dopamine remain active to preserve reward	Low dopamine activity are associated with cognitive dysfunction
Serotonin (5-HT)	Mixed effects: 5-HT stimulation of 5-HT_2_ receptors is anxiogenic.	Prefrontal cortex, amygdala, hippocampus.	High levels of cortisol decrease in 5-HT_1A_ receptors	High activity of postsynaptic 5-HT_1A_ receptors cause recovery	Low 5-HT_1A_ receptors may predispose to anxiety and depression
Benzodiazepine receptors	Acute stress	Prefrontal cortex, hippocampus	decreased 5-HT_1A_ and decreased benzodiazepine receptor function	Resistance to stress-induced down-regulation of benzodiazepine receptors	Decreased cortical benzodiazepine receptors cause panic disorder and PTSD
Testosterone	Stress-induced decrease in assertive behavior and increase in depression	Hypothalamus	CRH decreases testosterone levels	Increase in testosterone activate coping and reduce DEP.	Decreased CSF testosterone found in PTSD;
Estrogen	increases in estrogen damage (HPA) and NA	Hypothalamus, hippocampus	increases function of benzodiazepine, decreases function of 5-HT_1A_ receptors	estrogen may attenuate effects of stress-induced HPA axis and noradrenergic system activation	Long-term increases in estrogen down-regulate 5-HT_1A_ receptors and increase risk or depression.

**Table 2:** Summary of Neurobiology of PTSD

**Abnormalities in Sympathetic branch**
Severe stress causes dysfunction of locus coeruleus causing hypersensitivity to external stimuli
**Studies of combat veterans vs. non-combat veterans**
**Differences in combat veterans:**
Increased heart rate and anxiety during combat related stimuli
Increased urinary excretion of NE in PTSD patients
Abnormalities of the Hypothalamic Pituitary Adrenal Axis (HPA)
In acute /chronic stress and major depression: Cortisol levels increase
**Among non-combat veterans 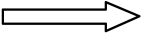 No change**
**In PTSD, sensitization of the HPA occurs:**
Cortisol levels are chronically decreased
Patients with PTSD show lower cortisol levels up to 50 years post trauma
Lower cortisol levels immediately after trauma, risk factor for development of PTSD later date
**Hipocampal MRI measurements smaller in PTSD**
Correlates with severity of trauma
Cognitive deficits
PTSD symptoms
**Functional neuro-imaging whilst traumatic material is shown**
Hyperactive amygdale
Anterior paralimbic regions activated
Decreased activity in anterior cingulate cortex
Decreased blood flow to hippocampus
**Summary Model of PTSD**
Amygdala and related structures are overactive
Also known as “hot and emotional” memory system
Hippocampus and anterior cingulated cortex is underactive
Also known as “cool and cognitive” memory system

## AUTHORS' CONTRIBUTIONS

AB: = Investigator and major contributor to writing of manuscript and graphics.
TJHC: = Editorial contributions and literature review;
MAM: = Major editorial contribution and co-author
JOB: = Clinical advisor and contributor to clinical issues and treatment
ALHC: = Contributor to hypothesis and overall manuscript review;
BWD: = Contributed to the preparation of the manuscript in terms of genetic aspects.
ERB: = Provided comments relative to clinical aspects and contributed to the preparation of the manuscript
SR: = Provided input to the original preparation of the manuscript and clinical input
MSG: = Provided comments relative to clinical and psychiatric aspects and contributed to the preparation of the manuscript
RLW: = Provided important literature information and editorial comments.
JG: = Provided clinical treatment and new diagnostic information and co—authored
SM: = Provided invaluable literature resources, clinical information and co-authored.
MK: = Provided literature input and editorial contribution and formatting
KB: = Co investigator and equally contributed to drafting of the manuscript to AB.
MO-B: = Contributor to writing and editing of the manuscipt

All authors read and approved the final manuscript.

## RESOURCE INFORMATION

The following Web site links are suggested for addional information http:// www.addictionsearch.com and http://www.drugstrategies.org; http://www.RDsyndrome.com
